# Obesity‐dependent dysregulation of glucose homeostasis in kinase suppressor of ras 2^*−*/*−*^ mice

**DOI:** 10.14814/phy2.12053

**Published:** 2014-07-04

**Authors:** MaLinda D. Henry, Diane L. Costanzo‐Garvey, Paula J. Klutho, Robert E. Lewis

**Affiliations:** 1 Eppley Institute for Research in Cancer and Allied Diseases, University of Nebraska Medical Center, Omaha, Nebraska; Division of Cardiovascular Medicine, Carver College of Medicine, University of Iowa, 285 Newton Road2270‐A CBRB, Iowa City, 52242, Iowa, USA

**Keywords:** AMPK, glucose metabolism, insulin resistance, KSR2, obesity

## Abstract

Disruption of KSR2 in humans and mice decreases metabolic rate and induces obesity, coincident with dysregulation of glucose homeostasis. Relative to wild‐type mice, *ksr2*^*−/−*^ mice are small prior to weaning with normal glucose tolerance at 6 weeks of age, but demonstrate excess adiposity by 9 weeks and glucose intolerance by 12–14 weeks. Defects in AICAR tolerance, a measure of whole‐body AMPK activation, are detectable only when *ksr2*^*−/−*^ mice are obese. Food restriction prevents the obesity of adult *ksr2*^*−/−*^ mice and normalizes glucose and AICAR sensitivity. Obesity and glucose intolerance return when ad lib feeding is restored to the diet‐restricted mice, indicating that glucose dysregulation is secondary to obesity in *ksr2*^*−/−*^ mice. The phenotype of C57BL/6 *ksr2*^*−/−*^ mice, including obesity and obesity‐related dysregulation of glucose homeostasis, recapitulates that of humans with KSR2 mutations, demonstrating the applicability of the C57BL/6 *ksr2*^*−/−*^ mouse model to the study of the pathogenesis of human disease. These data implicate KSR2 as a physiological regulator of glucose metabolism during development affecting energy sensing, insulin signaling, and lipid storage, and demonstrate the value of the C57BL/6 *ksr2*^*−/−*^ mouse model as a unique and relevant model system in which to develop and test therapeutic targets for the prevention and treatment of obesity, type 2 diabetes, and obesity‐related metabolic disorders.

## Abstract (precis)

A fraction of individuals with obesity‐induced insulin resistance and diabetes respond to diet with improved glucose metabolism. The phenotype of C57BL/6 *ksr2*^*−/−*^ mice, including obesity and obesity‐related dysregulation of glucose homeostasis, recapitulates that of humans with KSR2 mutations. These data show that the glucose intolerance and AICAR insensitivity that accompanies KSR2 disruption in mice is preventable or reversible by diet, suggesting that dietary intervention in humans with KSR2 mutations should have similar effects.

## Introduction

Obesity has become a prevalent and destructive health disorder in our society (Flier 2004) linked to some of the leading causes of preventable death including heart disease, stroke, certain types of cancer, and type 2 diabetes (NIH 1998). The prevalence of obesity and diabetes increases the need for in vivo models that reveal molecular pathways driving the pathogenesis of obesity and obesity‐related disorders. Three major physiological mechanisms produce obesity in rodents: hyperphagia, defective nonshivering thermogenesis, and the preferential deposition of calories into adipose tissue (Chua 1997). There are numerous examples (Yen et al. 1994; Zhang et al. 1994; Tecott et al. 1995; Chen et al. 1996; Lee et al. 1996; Ollmann et al. 1997; Balthasar et al. 2005; Fellmann et al. 2013) of rodent models that have been genetically engineered or developed from spontaneous mutations to investigate the contribution of a variety of signaling pathways to the regulation of these three mechanisms. For example, *ob/ob* and *db/db* mouse models were critical to identifying leptin and its receptor (Hummel et al. 1966; Zhang et al. 1994; Halaas et al. 1995; Maffei et al. 1995; Tartaglia et al. 1995; Lee et al. 1996), and identifying the JAK/STAT signaling pathway as the major mechanism through which leptin communicates satiety to the hypothalamus (Ghilardi et al. 1996; Bates et al. 2003). Thermogenic defects in *ob/ob* and *db/db* mice played an integral part in identifying the mechanisms through which leptin regulates nonshivering thermogenesis to affect organismal energetic homeostasis (Chua 1997). Subsequent identification that leptin and its receptor were mutated in rare forms of monogenic human obesity validated the importance of these models (Montague et al. 1997; Farooqi et al. 2007).

Mice with disrupted alleles of Kinase Suppressor of Ras 2 (KSR2) appear molecularly distinct from other genetic models as they become obese due to a lower metabolic rate without a decrease in locomotor activity (Costanzo‐Garvey et al. 2009). Mice lacking KSR2 are markedly insulin resistant in liver, muscle, and adipose depots. KSR2 is also mutated in a subpopulation of humans with early onset obesity (Pearce et al. 2013). Individuals carrying KSR2 mutations exhibit childhood hyperphagia, reduced basal metabolic rate, and severe insulin resistance. These observations reveal *ksr2*^*−/−*^ mice as a useful model for understanding physiological pathways contributing to human obesity and for revealing novel biochemical mechanisms that link obesity to insulin resistance.

KSR2 is a scaffold protein in the Raf/MEK/ERK signaling cascade, where it functions along with its paralog, KSR1, to coordinate the interaction of these molecules to facilitate signal transduction and regulate the intensity and duration of ERK signaling (Dougherty et al. 2009). KSR2 also interacts with and promotes activation of the primary regulator of cellular energy homeostasis, AMPK (Costanzo‐Garvey et al. 2009; Fernandez et al. 2012). In *ksr2*^*−/−*^ mice, decreased AMPK activation impairs the oxidation of fatty acids and increases their storage as triglycerides, promoting obesity and insulin resistance (Costanzo‐Garvey et al. 2009). Some KSR2 mutations in individuals with early onset obesity disrupt ERK activation or impair interaction of the scaffold with AMPK (Pearce et al. 2013). Together, these data implicate KSR2 as a potential sensor of cellular energy status and a key effector in whole‐body energy regulation in mice and humans.

KSR2 mutations in humans (Pearce et al. 2013) recapitulate the obesity and severe insulin resistance observed in C57BL/6 *ksr2*^*−/−*^ mice (Costanzo‐Garvey et al. 2009), revealing them as a disease‐relevant model system, allowing investigation into mechanisms through which KSR2‐dependent signaling may contribute to the onset and progression of obesity and diabetes in humans. Here, we demonstrate that C57BL/6 *ksr2*^*−/−*^ mice are not born with defects in glucose regulation, but that these defects arise as a result of obesity that develops later in the life of *ksr2*^*−/−*^ adults. We show that defects in glucose homeostasis can be prevented through postweaning food restriction of young *ksr2*^*−/−*^ mice, and reversed by restricting the diet of obese *ksr2*^*−/−*^ adults already suffering from glucose intolerance and insulin resistance. Given the similarities in metabolic characteristics between the C57BL/6 *ksr2*^*−/−*^ model and humans bearing KSR2 mutations, we propose the broad utility of *ksr2*^*−/−*^ mice to the study, treatment, and prevention of human obesity and obesity‐related disorders.

## Research Design and Methods

### Generation and housing of mice

DBA1/LacJ *ksr2*^*−/−*^ mice were as described previously (Costanzo‐Garvey et al. 2009). Mice were backcrossed 10 generations to generate C57BL/6 *ksr2*^*−/−*^ mice.

First‐generation offspring (F1) from breeding of heterozygous parents were used in these experiments. From this breeding 131 KSR2^+/+^, 181 KSR2^+/−^, and 77 KSR2^*−/−*^ mice (0.34:0.47:0.20) were generated, approximating the expected Medelian ratio of 0.25:0.50:0.25. The Institutional Animal Care and Use Committee (University of Nebraska Medical Center, Omaha, NE) approved all studies. Animals were maintained on a 12‐h light/dark schedule and had free access to laboratory chow (Harlan Teklad LM 485, Harlan Laboratories, Inc, Indianapolis, IN) and water except as described below.

### Food restriction of young, lean *ksr2*^*−/−*^ mice

For food restriction studies, female *ksr2*^*−/−*^ and age‐matched WT control mice were placed into one of four groups: (1) WT ad libitum (WT ad lib), (2) WT food restricted (WT FR), (3) *ksr2*^*−/−*^ ad libitum (*ksr2*^*−/−*^ ad lib), or (4) *ksr2*^*−/−*^ food restricted (*ksr2*^*−/−*^ FR). At 5 weeks of age, daily ad libitum food intake was determined for 1 week for each mouse by subtracting the weight of remaining chow from the weight of total chow provided. *ksr2*^*−/−*^ FR and WT FR mice received 70% of their daily ad libitum food intake once daily from 6 to 12 weeks of age. Following 6 weeks of food restriction, animals were subjected to glucose homeostasis tests (see glucose, insulin and 5‐aminoimidazole‐4‐carboxamide‐1‐*β*‐d‐ribofuranoside [AICAR] tolerance tests described below) during which time food restriction continued, totaling 8 weeks of food restriction. Following tests of glucose homeostasis, previously FR mice were allowed ad libitum access to food at 14 weeks of age. Daily food intake was again determined for these *ksr2*^*−/−*^ FR‐ad lib and WT FR‐ad lib mice as well as their ad lib fed controls. Animals were refed for 13 weeks. Glucose homeostasis testing was repeated on these 6‐month‐old FR‐ad lib and ad lib control animals.

### Food restriction of adult, obese *ksr2*^*−/−*^ mice

Female *ksr2*^*−/−*^ and age‐matched WT control mice were placed into one of four groups: (1) WT ad lib, (2) WT FR, (3) *ksr2*^*−/−*^ ad lib, or (4) *ksr2*^*−/−*^ FR. All mice were fed ad libitum from weaning until 16 weeks of age. Daily ad libitum food intake was determined for each mouse for 1 week starting at 15 weeks of age. At 16 weeks of age, WT FR and *ksr2*^*−/−*^ FR mice received 70% of their daily ad libitum food intake once daily from 16 to 22 weeks of age. WT ad lib and *ksr2*^*−/−*^ ad lib mice were fed ad libitum throughout the study.

### Dual Energy X‐ray Absorptiometry (DEXA)

Mice were weighed weekly on a digital scale. Quantification of lean mass, fat mass, and % body fat was performed every 2 weeks using dual energy X‐ray absorptiometry (DEXA). Mice were anesthetized using a mixture of inhaled isoflurane and oxygen (anesthetization using 3% isoflurane and 1 L/min oxygen; maintenance using 1–2% isoflurane and 1 L/min oxygen) and placed prone on the imaging positioning tray. Mice were scanned using a Lunar PIXImus^™^ densitometer (GE Medical‐Lunar, Madison, WI).

### Glucose, insulin, and 5‐aminoimidazole‐4‐carboxamide‐1‐*β*‐d‐ribofuranoside (AICAR) tolerance tests

To determine the role KSR2 plays in glucose homeostasis, glucose (GTT), insulin (ITT), and AICAR (ATT) tolerance tests were performed in *ksr2*^*−/−*^ and WT mice. The AICAR tolerance test measures whole‐body response to the activation of AMPK by the AMPK agonist AICAR (Viollet et al. 2003). GTT were performed after a 10‐h fast. ITT and ATT were performed following a 4‐h fast. Mice were injected intraperitoneally (IP) with D‐glucose (20% solution, 2 g/kg of body weight) for GTT, human insulin (1 unit/kg) for ITT, or AICAR (0.25 g/kg) for ATT. Blood glucose levels were determined following injection at the indicated times.

### Metabolite assays

Blood was collected by tail bleeds of live animals or via cardiac puncture of euthanized animals. Animals were fasted overnight for 10–12 h prior to collection for blood glucose and serum insulin measurement. Blood glucose was measured with an Ascensia Glucometer Elite (Bayer Corp., Elkhart, IN). For serum analysis, blood was allowed to clot at 4°C for 8–24 h, and the serum was separated by centrifugation for 10 min at 100 *g*. Serum was transferred to a new tube and stored at −20°C until assay. Serum insulin was measured with the Mouse Insulin ELISA Kit (ChrystalChem, Downers Grove, IL) using mouse standards. Serum leptin was measured with a Mouse Leptin ELISA kit (Millipore, Billerica, MA).

### Leptin responsiveness

Responsiveness to leptin was assessed by IP injecting 4‐ to 5‐month‐old *ksr2*^*−/−*^ and WT mice with 100 *μ*L phosphate‐buffered saline (PBS; as a control) followed 2 days later by an injection of 5 mg/kg leptin in 100 *μ*L and quantifying total food intake during the 24‐h period following each injection. Thus, each animal received two injections and served as its own control.

### Lipid quantification

Serum triglyceride and free fatty acid (FFA) levels were quantified from ad lib and FR WT and *ksr2*^*−/−*^ mice using Triglyceride Reagent (T2449; Sigma‐Aldrich, St. Louis, MO) and a Free Fatty Acids Half Micro Test (11383175001; Roche, Indianapolis, IN). Lipid accumulation in ad lib and FR WT and *ksr2*^*−/−*^ mice was visualized by hematoxylin and eosin staining of white adipose tissue (WAT) and brown adipose tissue (BAT) sections.

### Statistical analysis

GraphPad Prism version 5.04 for Windows (GraphPad Software, San Diego, CA) was used for graphics design and statistical analyses. To determine the extent to which disruption of KSR2 affected the indicated response variable at a single time point, we used a Student's *t*‐test, applying a Bonferroni adjustment when multiple pairwise comparisons were made (Sokal and Rohlf 1995). To determine the effect of KSR2 disruption on the indicated response variable over time or under various treatments, we applied a two‐way analysis of variance (ANOVA) with genotype and time, age, or treatment as dependent factors (Sokal and Rohlf 1995). When multiple data points were drawn sequentially from the same animal, pseudoreplication was avoided by performing a repeated measures two‐way ANOVA. When results from the two‐way ANOVA indicated that genotype had a significant effect, we performed an additional series of Bonferroni‐adjusted Student's *t*‐tests to identify the time point at which or treatment under which the effect of KSR2 disruption became significant. Data are shown as the mean ± standard deviation (SD). Significance was accepted at *P* < 0.05. Unless indicated otherwise for clarity, significant comparisons are represented as follows: *P < 0.05, **P < 0.01, ***P < 0.001, and ****P < 0.0001.

## Results

### C57BL/6 *ksr2*^*−/−*^ mice are small and lean at weaning, but become obese as adults

**Figure 1.**
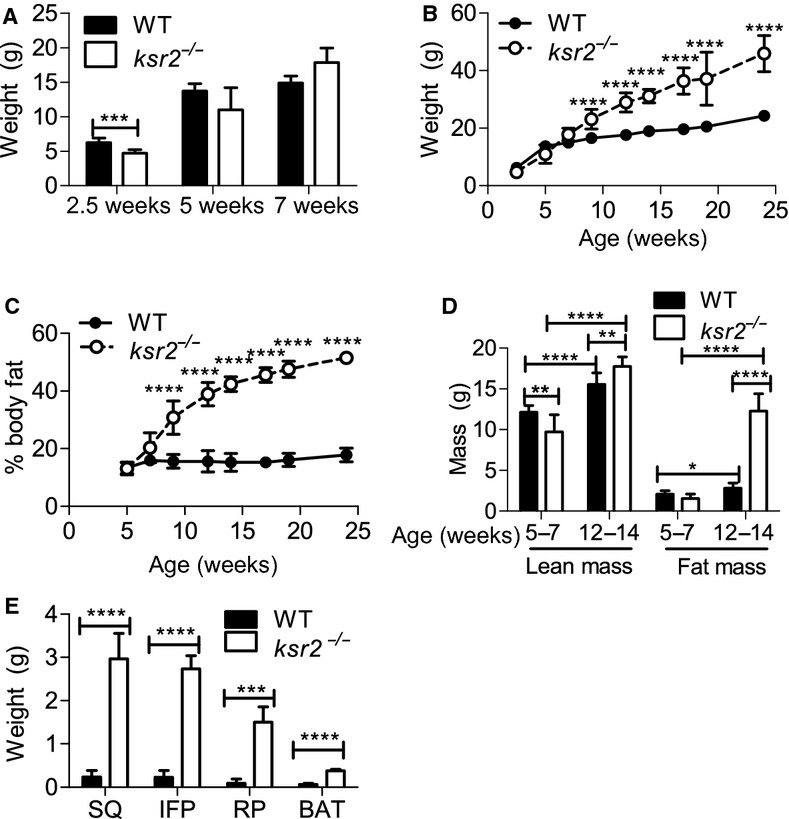
C57BL/6 *ksr2*^*−/−*^ mice are small and lean at weaning, but become obese as adults. (A) Body weights of WT and *ksr2*^*−/−*^ mice compared at 2.5 (*n* = 8–9), 5 (*n* = 3–5), and 7 (*n* = 3–5 per genotype) weeks of age. Body weights (B) and percentage body fat (C) determined by DEXA of WT and *ksr2*^*−/−*^ mice compared from 2.5 to 24 weeks of age. 2.5 weeks, *n* = 8–9; 5–7 weeks, *n* = 3–5; 9–14 weeks, *n* = 4–9; and 17–24 weeks, *n* = 2–4 per genotype. (D) Lean and fat mass determined by DEXA of WT and *ksr2*^*−/−*^ mice at 5–7 weeks of age (*n* = 8) and 12–14 weeks of age (*n* = 8–11 per genotype). (E) The weight of adipose distinct depots was determined in WT and *ksr2*^−/−^ mice. SQ, subcutaneous; IFP, inguinal fat pad; RP, retroperitoneal; BAT, brown adipose tissue. *P < 0.05, **P < 0.01, ***P < 0.001, and ****P < 0.0001.

We reported previously that DBA1/LacJ *ksr2*^*−/−*^ mice are obese (Costanzo‐Garvey et al. 2009). We generated and characterized congenic C57BL/6 *ksr2*^*−/−*^ mice. The C57BL/6 strain is commonly used to study the genetics and physiology of obesity and diabetes. At birth, *ksr2*^*−/−*^ mice are not distinguishable from WT littermates by size. However, at 2.5 weeks of age and while still nursing, *ksr2*^*−/−*^ neonates weigh 25% less than their WT littermates (Fig. 1A). After weaning, *ksr2*^*−/−*^ mice demonstrate accelerated growth rates that allow them to surpass their WT littermates in body weight by 9 weeks of age (Fig. 1B). This increase in weight is due primarily to an increase in fat mass (Fig. 1C and D). By 13 weeks of age *ksr2*^*−/−*^ mice have three times more fat mass than a WT mouse and a 10% increase in lean mass (Fig. 1D). All adipose depots in *ksr2*^−/−^ mice are increased relative to the same depots in WT mice (Fig. 1E).

### Defects in glucose homeostasis in *ksr2*^*−/−*^ mice are coincident with the development of obesity

**Figure 2.**
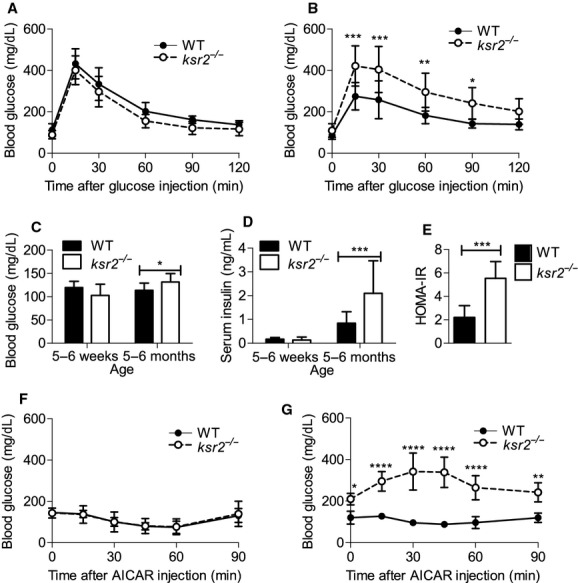
Defects in glucose homeostasis in *ksr2*^*−/−*^ mice are coincident with the development of obesity. GTT of (A) 6‐week‐old (WT *n* = 6; *ksr2*^*−/−*^ *n* = 4) and (B) 5‐month‐old (WT *n* = 8; *ksr2*^*−/−*^ *n* = 8) WT and *ksr2*^*−/−*^ mice. (C) Blood glucose (5–6 weeks old, *n* = 10; 5–6 months old, *n* = 10–13 per genotype) and (D) serum insulin levels (5–6 weeks, *n* = 8–9; 5–6 months old, *n* = 14–16 per genotype) of 5‐ to 6‐week‐old and 5‐ to 6‐month‐old fasted WT and *ksr2*^*−/−*^ mice. (E) HOMA‐IR, calculated from fasting glucose and insulin levels in 6‐month‐old WT and KSR2^−/−^ mice (n = 6–8 per genotype), revealed significant insulin resistance in the knockout mice. (F) ATT of 7‐week‐old (*n* = 5 per genotype) and (G) 5‐month‐old (*n* = 4 per genotype) WT and *ksr2*^*−/−*^ mice. *P < 0.05, **P < 0.01, ***P < 0.001, and ****P < 0.0001.

*ksr2*^*−/−*^ mice display many obesity‐related changes in glucose regulation including glucose and AICAR intolerance and high fasting blood glucose and insulin levels (Fig. 2A–G; Costanzo‐Garvey et al. 2009). To determine the extent to which glucose homeostasis was deregulated prior to the onset of obesity, 6‐ and 7‐week‐old *ksr2*^*−/−*^ mice were challenged with GTT and ATT prior to becoming obese. Six‐week‐old *ksr2*^*−/−*^ mice responded similar to WT mice when injected with glucose (Fig. 2A). In contrast, 5‐month‐old *ksr2*^*−/−*^ mice are glucose intolerant (Fig. 2B). In addition, fasting glucose and insulin levels are normal in 5‐ to 6‐week‐old *ksr2*^*−/−*^ mice, but elevated in 5‐ to 6‐month‐old *ksr2*^*−/−*^ mice (Fig. 2C and D). HOMA‐IR was calculated from the fasting level of glucose and insulin in 6‐month‐old WT and *ksr2*^−/−^ mice, revealing significant insulin resistance in the knockout mice (Fig. 2E). While 5‐month‐old *ksr2*^*−/−*^ mice respond to AICAR by inappropriately increasing their blood glucose (Fig. 2G), 7‐week‐old *ksr2*^*−/−*^ mice do not display a defect in AICAR tolerance when compared to WT mice of the same age (Fig. 2F). These data indicate that the dysregulation of glucose homeostasis is not present in younger *ksr2*^*−/−*^ mice but coincides with, or occurs subsequent to, rapid weight gain.

### Food restriction of young *ksr2*^*−/−*^ mice prevents obesity

**Figure 3.**
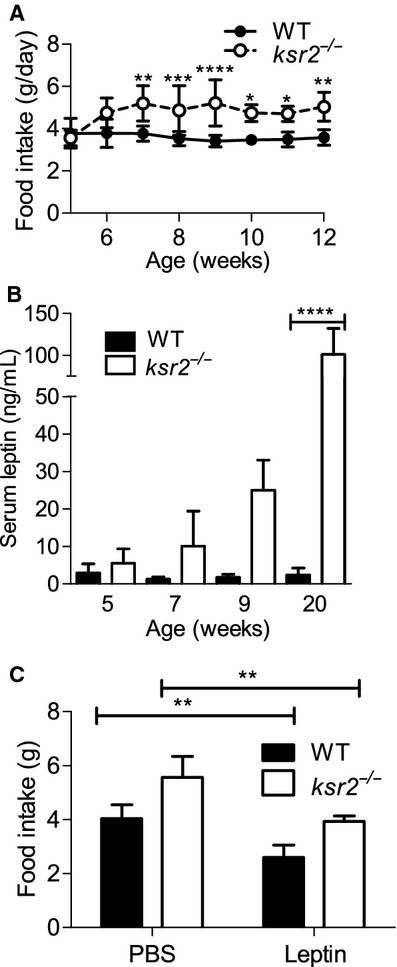
C57BL/6 *ksr2*^*−/−*^ mice are hyperphagic, but responsive to exogenous leptin. (A) Food intake of WT and *ksr2*^*−/−*^ mice at 5–12 weeks of age. Five to seven weeks, *n* = 3–5; 8–12 weeks, *n* = 5–9 per genotype. (B) Serum leptin levels of WT (*n* = 3–6) and *ksr2*^*−/−*^ (*n* = 4–5) mice at 5, 7, 9, and 20 weeks of age. (C) 24‐h food intake following IP injection of 100 *μ*L PBS or 100 *μ*L of 5 mg/kg leptin in 4‐ to 5‐month‐old WT and *ksr2*^*−/−*^ mice (*n* = 4 per genotype). *P < 0.05, **P < 0.01, ***P < 0.001, and ****P < 0.0001.

Contrary to DBA1/LacJ *ksr2*^*−/−*^ mice, which do not eat more than WT mice (Costanzo‐Garvey et al. 2009), C57BL/6 *ksr2*^*−/−*^ mice are hyperphagic (Fig. 3A). At 5 weeks of age C57BL/6 *ksr2*^*−/−*^ mice eat the same amount of food as WT mice, but by 7 weeks of age *ksr2*^*−/−*^ mice eat significantly more food per day than WT mice (Fig. 3A). Whereas lean 5‐week‐old *ksr2*^*−/−*^ mice have normal serum leptin levels, obese 5‐month‐old *ksr2*^*−/−*^ mice display high serum leptin levels in comparison to WT controls (Fig. 3B; Costanzo‐Garvey et al. 2009). DBA1/LacJ *ksr2*^*−/−*^ mice, which are normophagic, retained sensitivity to leptin administration (Costanzo‐Garvey et al. 2009). To determine if the hyperphagia present in C57BL/6 *ksr2*^*−/−*^ mice is due to leptin resistance, mice were injected with leptin and food intake monitored. Acute leptin administration suppressed food intake by 26% in *ksr2*^*−/−*^ mice and by 37% in WT mice (Fig. 3C). These data indicate that C57BL/6 *ksr2*^*−/−*^ mice, like DBA1/LacJ *ksr2*^*−/−*^ mice, remain responsive to leptin.

**Figure 4.**
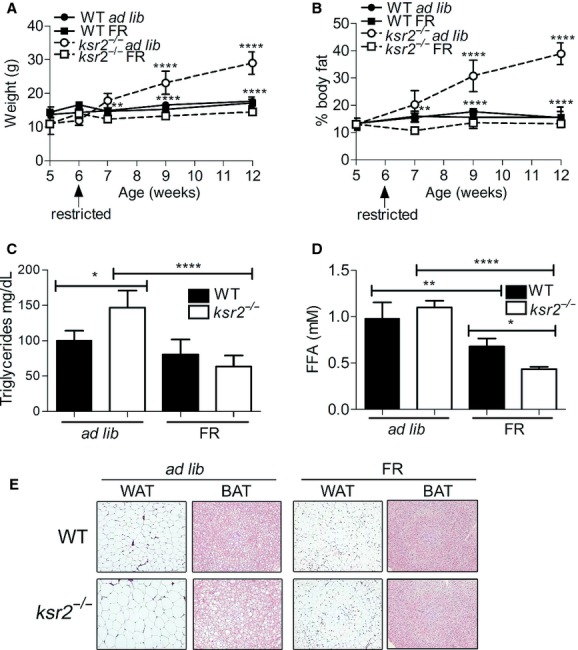
Food restriction of young *ksr2*^*−/−*^ mice prevents obesity. Body weight (A) and percentage body fat (B) of WT ad lib, WT FR,* ksr2*^*−/−*^ ad lib, and *ksr2*^*−/−*^ FR mice from 5 to 12 weeks of age. Ad lib 5–7 weeks, *n* = 3–5 per genotype; ad lib 8–12 weeks, *n* = 4–9 per genotype; FR 5–12 weeks, *n* = 4 per genotype. The top set of asterisks identifies significant comparisons between WT ad lib and *ksr2*^*−/−*^ ad lib mice. The bottom set of asterisks identifies significant comparisons between *ksr2*^*−/−*^ ad lib and *ksr2*^*−/−*^ FR mice. Serum triglyceride (C) and FFA (D) levels in 12‐ to 14‐week‐old WT ad lib, WT FR,* ksr2*^*−/−*^ ad lib, and *ksr2*^*−/−*^ FR mice (*n* = 4–5 per treatment). (E) Hematoxylin and eosin staining of WAT and BAT sections from 16‐week‐old ad lib and FR WT and *ksr2*^*−/−*^ mice. *P < 0.05, **P < 0.01, ***P < 0.001, and ****P < 0.0001.

Since weight gain and fat accumulation were coincident with hyperphagia in adult C57BL/6 *ksr2*^*−/−*^ mice, we sought to determine the extent to which food restriction in *ksr2*^*−/−*^ young mice would prevent or forestall obesity when they were adults. WT and *ksr2*^*−/−*^ mice were food restricted (FR) for 6 weeks. Food intake was measured at 5 weeks of age (3.95 g/day ± 0.21 for WT; 3.70 g/day ± 0.21 for *ksr2*^*−/−*^). Each FR mouse was fed 70% of their daily ad libitum amount from 6 to 12 weeks of age. Food restriction prevented obesity in *ksr2*^*−/−*^ mice (Fig. 4A and B). Although not statistically significant, *ksr2*^*−/−*^ FR mice tended to weigh less and be leaner than WT FR mice at 12 weeks of age (Fig. 4A and B), an age when *ksr2*^*−/−*^ mice fed an ad lib diet are significantly heavier than WT mice (Fig. 4A). In addition, food restriction in young *ksr2*^*−/−*^ mice normalized serum triglyceride levels in adult *ksr2*^*−/−*^ FR mice (Fig. 4C) and led to a significant reduction in FFA in the serum of adult *ksr2*^*−/−*^ FR mice compared to WT FR mice (Fig. 4D). Food restriction also led to a reduction in adipocyte cell size in WAT and BAT as well as to the reduction of lipid storage in BAT of *ksr2*^*−/−*^ mice compared to ad lib fed controls (Fig. 4E).

### Food restriction of young *ksr2*^*−/−*^ mice ameliorates defects in glucose regulation

**Figure 5.**
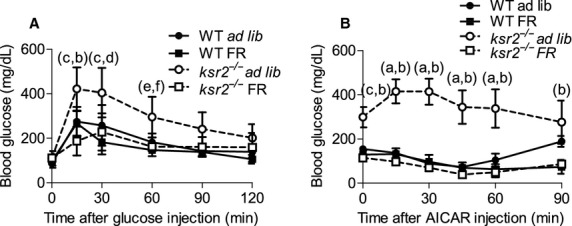
Food restriction of young *ksr2*^*−/−*^ mice ameliorates defects in glucose regulation. (A) GTT (ad lib, *n* = 8; FR,* n* = 4 per genotype) and (B) ATT (ad lib, *n* = 4; FR,* n* = 3–4 per genotype) of 12‐ to 14‐week‐old WT ad lib, WT FR,* ksr2*^*−/−*^ ad lib, and *ksr2*^*−/−*^ FR mice. a = *P* < 10^−5^; c = *P* < 10^−4^; e = *P* < 0.05 for comparisons between WT ad lib and *ksr2*^*−/−*^ ad lib mice. b = *P* < 10^−5^; d = *P* < 10^−4^; f = *P* < 0.05 for comparisons between *ksr2*^*−/−*^ ad lib and *ksr2*^*−/−*^ FR mice.

To determine what role KSR2 plays in glucose homeostasis, GTT and ATT were performed in 12‐ to 14‐week‐old food restricted *ksr2*^*−/−*^ and WT mice and compared to results in ad lib fed *ksr2*^*−/−*^ and WT controls. Food restriction improved glucose tolerance and AICAR sensitivity in *ksr2*^*−/−*^ mice (Fig. 5A–B). Food restricted *ksr2*^*−/−*^ mice responded to exogenous glucose or AICAR to the same degree as WT FR mice (Fig. 5A–B). These data suggest that the disrupted glucose homeostasis of *ksr2*^*−/−*^ mice is secondary to the obesity that results in these mice when fed ad libitum.

### Food restriction of young *ksr2*^*−/−*^ mice does not permanently alter glucose metabolism

**Figure 6.**
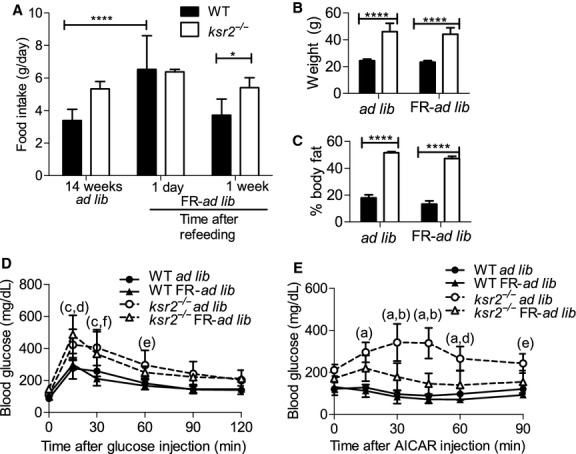
Food restriction of young *ksr2*^*−/−*^ mice does not permanently alter glucose metabolism. (A) WT (black bars, A–C) and *ksr2*^*−/−*^ (white bars, A–C) mice (fed ad lib, food restricted [FR], or food restricted then refed [FR‐ad lib]) at the indicated ages. Refeeding was performed in 14‐week‐old FR mice for 1 week. Fourteen‐week‐old ad lib (*n* = 8), 14‐week‐old FR‐ad lib 1 day after refeeding (*n* = 4), and 15‐week‐old FR‐ad lib (*n* = 4). Body weight (B) and percentage body fat (C) of 6‐month‐old WT and *ksr2*^*−/−*^ ad lib and FR‐ad lib fed mice (*n* = 4 per treatment). *P < 0.05, **P < 0.01, ***P < 0.001, and ****P < 0.0001. (D) GTT of 6‐month‐old WT ad lib (*n* = 8), WT FR‐ad lib (*n* = 4), *ksr2*^*−/−*^ ad lib (*n* = 8), and *ksr2*^*−/−*^ FR‐ad lib (*n* = 4) mice. a = *P* < 10^−5^; c = *P* < 10^−4^; e = *P* < 0.05 for comparisons between WT ad lib and *ksr2*^*−/−*^ ad lib mice. b = *P* < 10^−5^; d = *P* < 10^−3^; f = *P* < 0.05 for comparisons between WT FR‐ad lib and *ksr2*^*−/−*^ FR‐ad lib mice. (E) ATT of 6‐month‐old WT ad lib (*n* = 4), WT FR‐ad lib (*n* = 3), *ksr2*^*−/−*^ ad lib (*n* = 4), and *ksr2*^*−/−*^ FR‐ad lib (*n* = 4) mice. a = *P* < 10^−5^; c = *P* < 10^−4^; e = *P* < 0.05 for comparisons between WT ad lib and *ksr2*^*−/−*^ ad lib mice. b = *P* < 10^−5^; d = *P* < 10^−3^; f = *P* < 0.05 for comparisons between *ksr2*^*−/−*^ ad lib and *ksr2*^*−/−*^ FR‐ad lib mice.

To determine if food restriction resulted in permanent changes in glucose metabolism, mice were food restricted from 6 to 14 weeks of age, then allowed free access to food (FR‐ad lib). Immediately upon gaining free access to food, both WT and *ksr2*^*−/−*^ mice significantly increased their food intake (data not shown). However, after 1 week of free access to food, food intake in both WT and *ksr2*^*−/−*^ mice returned to levels observed in mice of the same age (14 weeks) that had not been restricted (Fig. 6A). While FR‐ad lib *ksr2*^*−/−*^ mice became once again hyperphagic compared to FR‐ad lib WT mice after 1 week of refeeding, there was no difference in food intake between FR‐ad lib *ksr2*^*−/−*^ mice and age‐matched ad lib *ksr2*^*−/−*^ controls (Fig. 6A). This result indicates that food restriction from weaning did not reprogram the set point of food intake in either *ksr2*^*−/−*^ or WT mice.

After 13 weeks of refeeding, 6‐month‐old FR‐ad lib *ksr2*^*−/−*^ and WT mice had similar weights compared to their respective ad lib fed controls (Fig. 6B). FR‐ad lib *ksr2*^*−/−*^ mice became obese and had similar body composition to *ksr2*^*−/−*^ ad lib fed controls (Fig. 6C). Although food restriction delayed obesity in *ksr2*^*−/−*^ mice, upon ad lib refeeding they regained weight quickly, most likely due to their persistent hyperphagia on the C57BL/6 background (Figs. 3A and 6A; Revelli et al. 2011) and decreased metabolic rate (Costanzo‐Garvey et al. 2009; Pearce et al. 2013).

We next tested the effect of food restriction followed by free access to food on glucose tolerance and AICAR sensitivity. FR‐ad lib WT mice had glucose tolerance indistinguishable from ad lib WT mice (Fig. 6D). Importantly, after FR, ad lib feeding restored glucose intolerance in *ksr2*^*−/−*^ mice (Fig. 6D). Although obesity for FR‐ad lib *ksr2*^*−/−*^ mice was delayed, the same defects in glucose handling observed in ad lib *ksr2*^*−/−*^ mice returned to FR‐ad lib *ksr2*^*−/−*^ mice once they became obese.

AICAR tolerance was not altered between FR‐ad lib and ad lib groups in WT mice (Fig. 6E). In *ksr2*^*−/−*^ mice, food restriction followed by free access to food did significantly improve AICAR tolerance, although FR‐ad lib *ksr2*^*−/−*^ mice remained somewhat AICAR insensitive compared to WT controls (Fig. 6E). However, the degree of insensitivity was less than what was detected in ad lib *ksr2*^*−/−*^ mice (Fig. 6E). This result may indicate that AICAR tolerance in adult *ksr2*^*−/−*^ mice is affected by food restriction, or it may reflect the fact that *ksr2*^*−/−*^ mice need to be obese for a longer length of time before AICAR insensitivity develops fully.

### Food restriction in adult *ksr2*^*−/−*^ mice reduces obesity

**Figure 7.**
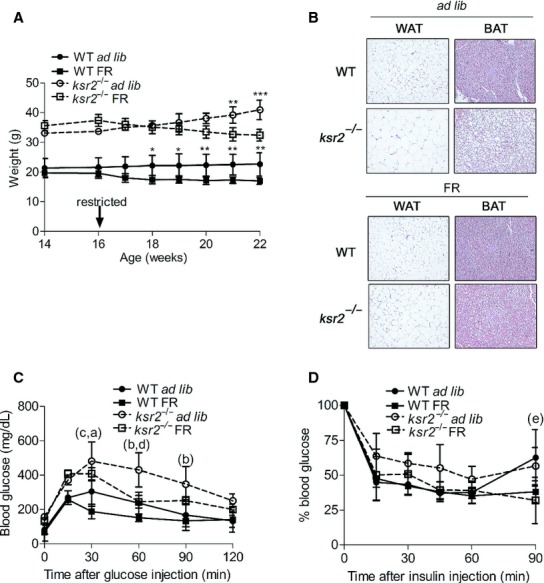
Food restriction in adult *ksr2*^*−/−*^ mice reduces obesity and improves glucose tolerance and insulin resistance. (A) Body weight of WT ad lib (*n* = 3) and *ksr2*^*−/−*^ ad lib (*n* = 3) mice compared to WT FR (*n* = 6) and *ksr2*^*−/−*^ FR (*n* = 4) mice, respectively, in response to food restriction from 16 to 22 weeks of age. The top set of asterisks identifies significant comparisons between *ksr2*^*−/−*^ ad lib and *ksr2*^*−/−*^ FR mice. The bottom set of asterisks identifies significant comparisons between WT ad lib and WT FR mice. *P < 0.05, **P < 0.01, ***P < 0.001. (B) Hematoxylin and eosin staining of WAT and BAT sections from 6‐month‐old ad lib and FR WT and *ksr2*^*−/−*^ mice. GTT (C) (*n* = 3–4 per treatment) and ITT expressed as a percentage of starting blood glucose levels (D) (*n* = 4–5 per treatment) of 6‐month‐old WT and *ksr2*^*−/−*^ ad lib, and FR mice. b = *P* < 10^−3^; c = *P* < 0.05 for comparisons between WT ad lib and *ksr2*^*−/−*^ ad lib mice. d = *P* < 10^−3^; e = *P* < 0.05 for comparisons between *ksr2*^*−/−*^ ad lib and *ksr2*^*−/−*^ FR mice. a = *P* < 10^−4^; f = *P* < 0.05 for comparisons between WT FR and *ksr2*^*−/−*^ FR mice.

To determine whether adult obesity and dysregulation of glucose homeostasis is reversible in adult *ksr2*^*−/−*^ mice, the diet of 16‐week‐old *ksr2*^*−/−*^ and WT mice was restricted to 70% of their daily food intake for 6 weeks. Adult WT and *ksr2*^*−/−*^ mice lost weight under food restriction (Fig. 7A). Weight loss in food restricted adult *ksr2*^*−/−*^ mice, but not WT mice, was visible as a significant reduction in adipocyte size in WAT and decreased lipid accumulation in BAT when compared to ad lib controls (Fig. 7B).

### Food restriction improves glucose tolerance and insulin resistance in obese adult *ksr2*^*−/−*^ mice

Food restriction improved glucose tolerance and insulin sensitivity in both *ksr2*^*−/−*^ and WT adult mice. Following food restriction, *ksr2*^*−/−*^ mice showed improved glucose tolerance relative to ad libitum fed *ksr2*^*−/−*^ mice, although blood glucose levels of FR *ksr2*^*−/−*^ mice remained higher than both FR and ad lib WT mice (Fig. 7C). Similarly, exogenous insulin modestly lowered blood glucose in food restricted adult *ksr2*^*−/−*^ mice relative to *ksr2*^*−/−*^ adults fed ad lib (Fig. 7D).

## Discussion

These data demonstrate that the dysregulated glucose homeostasis of *ksr2*^*−/−*^ mice is secondary to their obesity and can be prevented by dietary restriction prior to weaning or ameliorated by dietary restriction in obese adult knockout mice. Diet does not reverse the underlying metabolic defects caused by disruption of KSR2, however, as ad libitum feeding of diet‐restricted *ksr2*^*−/−*^ mice restores obesity and glucose intolerance. These data likely have significance for human obesity and obesity‐dependent insulin resistance, as KSR2 mutations in humans lower resting metabolic rate and promote obesity with severe insulin resistance (Pearce et al. 2013). The phenotype exhibited by C57BL/6 *ksr2*^*−/−*^ adult mice models a classic developmental pattern associated with an increased risk of type 2 diabetes including low neonatal body mass, accelerated rates of postnatal growth, and subsequent obesity leading to progressively worsening glucose intolerance and insulin resistance. Human infants with low birthweight or with poor postnatal nutrition have a higher risk of becoming obese as adults and developing metabolic syndrome (Barker 2005; Jimenez‐Chillaron et al. 2005; Bieswal et al. 2006; Isganaitis et al. 2009; Fernandez‐Twinn and Ozanne 2010). C57BL/6 *ksr2*^*−/−*^ mice are small at weaning, but quickly gain weight, surpassing WT controls by 9 weeks of age to become obese as adults primarily due to an increase in fat mass and lipid accumulation. Defects in glucose regulation, including glucose intolerance and insulin resistance, arise as a consequence of morbid obesity that develops later in life in C57BL/6 *ksr2*^*−/−*^ adults.

DBA1/LacJ *ksr2*^*−/−*^ mice consume less food than WT mice (Costanzo‐Garvey et al. 2009), whereas C57BL/6 *ksr2*^*−/−*^ mice are hyperphagic (Figs 3A and 6A; Revelli et al. 2011). Both DBA1/LacJ (Costanzo‐Garvey et al. 2009) and C57BL/6 (Fig. 1B–D) *ksr2*^*−/−*^ mice become obese. Pair‐feeding *ksr2*^*−/−*^ mice on a mixed 129SvEvBrd/C57BL/6J background reduces, but does not eliminate, obesity (Pearce et al. 2013), demonstrating that their obesity is not dependent on overeating. However, the hyperphagia of C57BL/6 *ksr2*^*−/−*^ mice likely explains why they become more obese than do DBA1/LacJ *ksr2*^*−/−*^ mice (Costanzo‐Garvey et al. 2009). Correspondingly, *ksr2*^*−/−*^ mice in the C57BL/6 background suffer from more extreme glucose and AICAR intolerance as well as more severe insulin resistance than DBA1/LacJ *ksr2*^*−/−*^ mice. Revelli et al. (2011) found *ksr2*^*−/−*^ mice on a mixed 129SvEvBrd/C57BL/6J background to be hyperphagic and leptin resistant. We observe that *ksr2*^*−/−*^ mice in both DBA1/LacJ and C57BL/6 backgrounds respond comparably to WT mice when treated acutely with exogenous leptin (Costanzo‐Garvey et al. 2009; Fig. 3C). The difference may be accounted for by the ability of leptin to suppress overnight feeding in this study, but not the appetite generated in *ksr2*^*−/−*^ mice pair fed for 2 weeks (Revelli et al. 2011). The responsiveness of C57BL/6 *ksr2*^*−/−*^ mice to leptin distinguishes them from murine models of obesity including the *ob/ob* mouse, *db/db* mouse, SHROB rat, JCR:LA‐cp rat, Zucker rat, and ZDF rat model systems (Fellmann et al. 2013). Given the rarity of cases of human obesity and obesity‐related disorders attributable to mutations in the leptin gene or receptor (Montague et al. 1997; Farooqi et al. 2002, 2007; Gibson et al. 2004; Farooqi and O'Rahilly 2006), the fact that the more common form of leptin resistance that results as a consequence of obesity is reversible in humans (Fellmann et al. 2013), and the demonstration that KSR2 mutations promote obesity in humans (Pearce et al. 2013), these data reveal C57BL/6 *ksr2*^*−/−*^ mice as a unique model system for identifying new effectors relevant to understanding the pathogenesis of human obesity and obesity‐related disorders. Furthermore, these mice may be useful in designing novel therapies for reducing obesity in humans.

Critical to the dissection of KSR2‐dependent signaling pathways is the identification of the tissues in which KSR2 acts to affect whole‐body energy balance. We recently identified a direct interaction between KSR2 and the AMP‐regulated kinase AMPK that is required for full activation of this cellular energy sensor (Costanzo‐Garvey et al. 2009; Fernandez et al. 2012). We proposed that defects in AMPK activation resulting from KSR2 disruption promoted adiposity by disabling acetyl CoA carboxylase regulation leading to the suppression of fatty acid metabolism and the promotion of triglyceride storage in *ksr2*^*−/−*^ mice. However, KSR2 mRNA expression in white adipose tissue is exceedingly low (Costanzo‐Garvey et al. 2009; Guo et al. 2014), suggesting that, in vivo, noncell autonomous effects of KSR2 on lipid metabolism predominate. Data here show that defects in AICAR tolerance, which presumably reflect the activation of AMPK in vivo, are not evident prior to increased adiposity in *ksr2*^*−/−*^ mice at 7 weeks of age (Fig. 2F). These data raise the possibility that KSR2‐mediated effects on AMPK function are regulated developmentally.

KSR2 is highly expressed in all areas of the brain, including the hypothalamus (Costanzo‐Garvey et al. 2009; Pearce et al. 2013), which serves as the main energy sensor for the body, regulating adaptive thermogenesis, food intake, and energy expenditure (Lowell and Spiegelman 2000; Lindsley and Rutter 2004; Herman and Kahn 2006). Thus, KSR2 in the hypothalamus may exert noncell autonomous effects on whole‐body energy balance. The hypothalamus contains two distinct neuron populations, those that secrete proopiomelanocortin (POMC) and cocaine‐amphetamine‐related transcript (CART) and those that secrete neuropeptide Y (NPY) and agouti‐related protein (AgRP). POMC and CART are anorexigenic and increase energy expenditure when stimulated, while NPY and AgRP are orexigenic (Cota et al. 2007). AMPK is an important signaling molecule in both neuronal types, responding to glucose levels as well as multiple hormones such as leptin, ghrelin, and adiponectin to regulate both food intake and energy expenditure. Deletion of AMPK*α*2 from POMC neurons results in obese mice with reduced energy expenditure and increased food intake, but which remain sensitive to leptin (Claret et al. 2007). Thus, our data suggest a role for KSR2 in the development or propagation of signals critical to the proper sensing of whole‐body energy status in the hypothalamus by potentially directing the subcellular distribution and activity of AMPK. Targeted disruption of KSR2 in the hypothalamus should reveal its potential role as a central regulator of energy sensing.

KSR2 is also detectable in skeletal muscle and liver (Guo et al. 2014). Thus, while noncell autonomous effects of KSR2 on energy balance may be mediated through the central nervous system, the potential remains for cell autonomous actions of KSR2 in muscle and liver that affect glucose homeostasis. Obese *ksr2*^−/−^ mice show hepatic steatosis (Costanzo‐Garvey and Lewis, unpublished). In combination with the defective activation of AMPK of adult *ksr2*^−/−^ mice, this observation is consistent with recent observations that AMPK protects against hepatic steatosis by phosphorylating SREBP‐1c, suppressing its cleavage and nuclear translocation, and repressing expression of SREBP‐1c target genes that promote lipogenesis and lipid accumulation (Li et al. 2011). Similarly, sedentary mice expressing dominant negative AMPK catalytic subunits in skeletal muscle (Mu et al. 2003) and *ksr2*^−/−^ mice (Costanzo‐Garvey et al. 2009) show a similar depletion of glycogen in the gastrocnemius. Tissue‐specific deletion of KSR2 in these tissues may reveal key organs contributing to metabolic control by KSR2.

The observation that *ksr2*^*−/−*^ mice recapitulate key metabolic characteristics identified in humans bearing mutations in KSR2 (Pearce et al. 2013) creates new opportunities for defining critical molecular mechanisms that control energy balance and for revealing their sites of action. Future studies should also reveal opportunities for early detection and prevention of obesity and its attendant metabolic disorders.

## Acknowledgments

The authors thank Dr. Stephen Bonasera for the training and use of the indirect calorimeter and DEXA equipment. Members of the Lewis laboratory are thanked for their helpful comments and criticism.

## Conflict of Interest

No potential conflicts of interest relevant to this article were reported.
